# The Influence of Land Use Intensity on the Plant-Associated Microbiome of *Dactylis glomerata* L.

**DOI:** 10.3389/fpls.2017.00930

**Published:** 2017-06-21

**Authors:** Jennifer Estendorfer, Barbara Stempfhuber, Paula Haury, Gisle Vestergaard, Matthias C. Rillig, Jasmin Joshi, Peter Schröder, Michael Schloter

**Affiliations:** ^1^Research Unit Comparative Microbiome Analysis, Helmholtz Zentrum MünchenNeuherberg, Germany; ^2^Institute for Biology, Freie Universität BerlinBerlin, Germany; ^3^Biodiversity Research/Systematic Botany, Institute for Biochemistry und Biology, University of PotsdamPotsdam, Germany; ^4^Chair of Soil Science, Technical University of MunichFreising, Germany

**Keywords:** *Dactylis glomerata*, land use change, endophytes, rhizosphere, soil microbiome, biodiversity

## Abstract

In this study, we investigated the impact of different land use intensities (LUI) on the root-associated microbiome of *Dactylis glomerata* (orchardgrass). For this purpose, eight sampling sites with different land use intensity levels but comparable soil properties were selected in the southwest of Germany. Experimental plots covered land use levels from natural grassland up to intensively managed meadows. We used 16S rRNA gene based barcoding to assess the plant-associated community structure in the endosphere, rhizosphere and bulk soil of *D. glomerata*. Samples were taken at the reproductive stage of the plant in early summer. Our data indicated that roots harbor a distinct bacterial community, which clearly differed from the microbiome of the rhizosphere and bulk soil. Our results revealed *Pseudomonadaceae, Enterobacteriaceae* and *Comamonadaceae* as the most abundant endophytes independently of land use intensity. Rhizosphere and bulk soil were dominated also by *Proteobacteria*, but the most abundant families differed from those obtained from root samples. In the soil, the effect of land use intensity was more pronounced compared to root endophytes leading to a clearly distinct pattern of bacterial communities under different LUI from rhizosphere and bulk soil vs. endophytes. Overall, a change of community structure on the plant–soil interface was observed, as the number of shared OTUs between all three compartments investigated increased with decreasing land use intensity. Thus, our findings suggest a stronger interaction of the plant with its surrounding soil under low land use intensity. Furthermore, the amount and quality of available nitrogen was identified as a major driver for shifts in the microbiome structure in all compartments.

## Introduction

Numerous studies have shown that healthy plants can host up to a few thousand different microbial species in their different organs, including roots, stems and leaves (Berendsen et al., 2012; Bulgarelli et al., 2012; Hacquard et al., 2015). Plant-associated microbes benefit from their host, as plants provide easily degradable carbon and a protected environment as well as physical structures for colonization (Kowalchuk et al., 2002). Vice versa, the plant-associated microbiome positively influences plant growth by supplying nutrients like nitrogen or phosphorus, improving stress tolerance and protecting plants from phytopathogens (Martinez-Viveros et al., 2010; Berendsen et al., 2012; Gaiero et al., 2013; Reinhold-Hurek et al., 2015).

The various plant compartments colonized by microbes do not provide constant environments, but change with plant developmental stage or as a result of environmental factors including temperature, water content, or nutrient availability (Kowalchuk et al., 2002; Tuteja and Sopory, 2008; Philippot et al., 2013). The importance of environmental factors as drivers for the plant phenotype has been confirmed in many studies, where the same plant genotype developed different phenotypes in response to differing environmental conditions (e.g., Schlichting, 1986; Sultan, 2000; Valladares et al., 2007), which in turn also favored the development of distinct microbial communities colonizing plant compartments (Compant et al., 2005; Gnanamanickam, 2006; Ernebjerg and Kishony, 2012; Gaiero et al., 2013; Tardieu, 2013; He et al., 2014). However, most of these studies were performed under greenhouse conditions, where key factors influencing plant performance were kept in the optimum and only one factor was changed. Wagner et al. (2016) postulated that the plant genotype plays only a minor role in the establishment of root-associated microbiomes under natural conditions in contrast to greenhouse experiments. Thus studies under natural conditions are needed as a next step to evaluate the importance of various multifactorial scenarios for the plant phenotype and plant-associated microbiomes.

Land use intensity strongly influences a large number of environmental factors including the amount and quality of nutrients, the soil structure and general biodiversity pattern (Birkhofer et al., 2012). Thus, land use intensification is a typical example for a driver inducing multifactorial changes in a given plant phenotype (Kirkham et al., 1996; Wedin and Tilman, 1996) and may consequently strongly modulate plant-associated microbial communities. Therefore, in this study we investigated the impact of different levels of land use intensity in grassland ecosystems on the root-associated microbiome of orchardgrass (*Dactylis glomerata* L.), a perennial forage grass which occurs under a wide range of land use intensity levels at grassland sides of Central Europe. Root-associated microbiomes were separated into (a) root endophytes and (b) microbes colonizing the rhizosphere. Furthermore, we investigated the impact of different levels of land use intensity on the microbiome of the bulk soil (c). We postulated that any effects of land use intensity will be most pronounced in the bulk soil and in the rhizosphere, whereas effects on root endophytes would be less pronounced and mainly modulated by the performance of the plants. We further hypothesized that in all investigated compartments land use intensity is negatively correlated with microbial diversity.

## Materials and Methods

### Sites Description and Sample Collection

This study was performed within the framework of the German Biodiversity Exploratories, a project investigating large-scale and long-term relationships of biodiversity and land use in Central European grasslands (Fischer et al., 2010). The Biodiversity Exploratory “Schwäbische Alb,” is located in the low mountain ranges in the southwest of Germany and covers an area of about 422 km^2^ including 100 grassland and forest experimental plots with different land use intensities (LUI). Plots are classified along a land use intensity index according to Blüthgen et al. (2012). As the index is based on three different management components (fertilization intensity, mowing frequency and grazing level) which are equally weighted, it provides a unique design for detailed analysis of long term effects on biota, as inter-annual variations in management practices are normalized. Eight plots were selected for this study. Mowing regime, fertilization and grazing management of these plots ranged from unfertilized meadows and pastures to highly fertilized meadows and mown pastures, resulting in a classification of four plots with high and four plots with low land use intensity, which were treated as true replicates (Plot IDs intensive LUI: AEG6, AEG19, AEG20, AEG21; Plot IDs extensive LUI: AEG7, AEG28, AEG33, AEG34; see Supplementary Table S1). The soil type of all plots has been characterized as Rendzic Leptosol (according to the FAO classification system). Mean annual temperature in the region is 9.9°C and mean annual precipitation is in the range of 730 mm.

Nine plants of *D. glomerata* per plot were collected in an area of 1.5 m × 1.5 m in June 2015 and pooled into three replicates to obtain enough material for further analyses (3 plants per replicate, that were closest together). All selected plants were in the reproductive state (production of seeds) without any disease symptoms like leaf spots. Plants were excavated carefully with adhering rhizosphere and bulk soil. Soil which could be shaken off easily from the roots was defined as bulk soil. The remaining soil that firmly adhered to the root was defined as rhizosphere. Bulk soil and root samples with adhering rhizosphere were collected in 15 ml tubes for DNA extraction, immediately frozen on dry ice and stored at -80°C until further analyzed. Above-ground biomass of the plant was harvested by cutting leaves with sterilized scissors and frozen immediately (-20°C). Additional bulk soil samples were stored at 4°C and sieved for soil carbon/nitrogen and dry weight measurement.

### Soil and Plant Carbon and Nitrogen Content

Dissolved carbon and nitrogen content in soil was measured after extraction of 5.0 g fresh soil using 0.01 M calcium chloride after shaking on a horizontal shaker for 45 min followed by centrifugation (2 min at 4500 x *g*). The extracts were filtered through a Millex HV Millipore filter, pore size, 0.45 μm (Merck, Darmstadt, Germany). Finally, water extractable organic carbon (WEOC) and nitrogen (WEON) were measured on DIMA-TOC 100 (Dima Tec, Langenhagen, Germany). Nitrate (NO_3_^-^-N) and ammonium (NH_4_^+^-N) were determined by continuous flow analysis using a photometric autoanalyzer (CFA-SAN Plus; Skalar Analytik, Germany).

Above-ground plant material was dried at 65°C for 2 days prior to pulverization in a Tissue LyserII (Qiagen GmbH, Germany). About 1.5 mg of the pulverized material was weighted into 3.5 mm × 5 mm tin capsules (HEKAtech GmbH, Wegberg, Germany). Total carbon and nitrogen contents were determined using an Elemental-Analysator ‘Euro-EA’ (Eurovector, Milano, Italy).

### Separation of Microbes from the Rhizosphere and Root Interior

*Dactylis glomerata* roots were transferred to 15 ml falcon tubes containing 7.5 ml sterile 1× PBS amended with 0.02% Silwet (PBS, AppliChem, Darmstadt; Silwet L-77) and shaken at 180 rpm for 5 min to remove the rhizosphere soil. This step was repeated three times by transferring the roots to new 15 ml falcon tubes containing sterile 1× PBS-S. The PBS-S buffer was subsequently centrifuged and the resulting pellets were frozen in liquid nitrogen and stored at -80°C.

After rhizosphere removal, roots were submerged in sterile 1% Tween 20 for 2 min, washed in sterile water, incubated for 2 min in 70% ethanol and subsequently rinsed three times with sterile distilled water. Afterward, roots were surface sterilized using 5% sodium hypochlorite for 10 min, washed eight times with sterile distilled water, frozen in liquid nitrogen and stored at -80°C. The disinfection efficiency was confirmed by the absence of PCR products after amplification of the 16S rRNA genes using DNA extracted from the final wash water as a template (data not shown). Additionally, no colonies were obtained when 200 μl of the final wash water were plated onto NB-agar plates and incubated for 10 days on 28°C (data not shown).

### Nucleic Acid Extraction

DNA was extracted using a phenol-chloroform-based method according to Lueders et al. (2004). We used surface sterilized roots, rhizosphere- and bulk soil samples taken from eight plots (3 replicates per plot) resulting in 72 samples in total. Briefly, root samples were frozen in liquid nitrogen and pre-homogenized in a TissueLyser II (Qiagen GmbH, Germany) prior to DNA extraction. 0.1 g of roots and 0.3 g of rhizosphere- and bulk soil, respectively, were homogenized in lysing matrix tubes E (MP Biomedicals, France) in 120 mM sodium phosphate buffer (pH 8) and TNS solution [500 mM Tris–HCl pH 8.0, 100 mM NaCl, 10 % SDS (wt/vol)] and centrifuged at 16,100 × *g* for 10 min at 4°C. The supernatant was removed and successively mixed with an equal amount of Phenol/Chloroform/Isoamyl Alcohol (25:24:1, Sigma–Aldrich) and Chloroform/Isoamyl Alcohol [(24:1 (vol/vol)] and centrifuged for 5 min at 16,100 × *g*. The DNA was precipitated using 30% (wt/vol) polyethylene glycol (PEG) solution [PEG 6000, NaCl], followed by 2 h of incubation on ice. After centrifugation (16,100 × *g*, 10 min, 4°C), the pellet was washed in ice-cold DNase/RNase free 70% ethanol, air-dried, and eluted in 30 μl 0.1% diethylpyrocarbonate water. The DNA concentration was quantified in duplicates using the Quant-iTPico^TM^ Green^®^ ds DNA assay Kit (Invitrogen, Carlsbad, CA, United States) according to the manufacturer’s instructions. Measurements were performed with a SpectraMax Gemini EM Fluorescence Plate Reader Spectrometer (Molecular Devices, Sunnyvale, CA, United States). Values were corrected for background fluorescence by addition of negative controls. DNA extracts were stored at -80°C until further use.

### PCR Amplification and Illumina Sequencing

Next generation sequencing was performed using the Illumina MiSeq platform (Illumina Inc., San Diego, CA, United States). Library preparation was accomplished according to the “16S Metagenomic Sequencing Library Preparation” protocol proposed by Illumina Inc., San Diego, CA, United States. Briefly, polymerase chain reaction (PCR) of the 16S rRNA region was performed in triplicates using the primers 335Fc (5′-CADACTCCTACGGGAGGC-3′) and 769Rc (5′-ATCCTGTTTGMTMCCCVCRC-3′) published by Dorn-In et al. (2015) with Illumina adapter sequences. The reaction mix contained of 12.5 μl NEB Next High Fidelity Master Mix (Illumina Inc., United States), 0.5 μl of each primer (10 pmol/μl), 2.5 μl of 3% BSA, 100–200 ng of template DNA and ad DEPC water 25 μl. PCR conditions were the following: 98°C for 5 min, followed by 20 cycles for rhizosphere and bulk soil samples and 28 cycles for roots samples, respectively, at 98°C for 10 s, 60°C for 30 s and 72°C for 30 s, followed by 72°C for 5 min. Each PCR was performed in triplicates to minimize PCR errors. Negative control samples using DEPC water instead of template DNA were treated similarly. DNA amplicons were analyzed using a 2% agarose gel, triplicates were pooled and subsequently purified using the Agencourt^®^AMPure^®^XP (Beckman Coulter Company, United States) extraction kit according to manufacturer’s instructions. However, the ratio of AMPure XP to PCR reaction was adapted to 0.6 to 1. Amplicon sizes and presence of primer-dimers were checked using a Bioanalyzer 2100 (Agilent Technologies, United States), the DNA 7500 kit (Agilent Technologies, United States) and quantified using the Quant-iT PicoGreen kit (Life Technologies, United States). Afterward, indexing PCR was performed using 12.5 μl NEB Next High Fidelity Master Mix, 10 ng DNA of the previous PCR products and 10 pmol of each primer containing adapter overhangs. For each indexing PCR the annealing temperature was reduced to 55°C and the number of cycles set to eight cycles. Purified PCR products were pooled in equimolar ratios to a final concentration of 4 nM and sequenced using the MiSeq Reagent kit v3 (600 cycles) (Illumina Inc., United States) for paired-end sequencing. Sequence files were deposited in the NCBI Sequence Read Archive under accession number SRP102620.

### Sequence Data Analysis

Sequences were analyzed using QIIME (Quantitative Insights into Microbial Ecology) software package version 1.9.1 (Caporaso et al., 2010) and default parameters. FASTQ files were trimmed and merged with a minimum read length of 50 and minimum Phred score of 15 using AdapterRemoval (Schubert et al., 2016). After removal of chimeric sequences, quality and length filtering (400–480 bp) of the merged reads with DeconSeq (Schmieder and Edwards, 2011), sequences were clustered into operational taxonomic units (OTUs) at 97% sequence identity with an open reference strategy using the GreenGenes database (13_5 release) as a reference (DeSantis et al., 2006). Subsequently, taxonomic classification was carried out using RDP classifier (Wang et al., 2007) retrained on the GreenGenes 16S rRNA reference database. The output was filtered with an abundance cut-off of 0.005% to remove singletons. A core set of QIIME diversity analyses was run on rarefied data for categories to compare (compartment and land use intensity). Faith’s phylogenetic diversity was used as a measure for α-diversity. Statistical significance in the global bacterial community composition was determined by permutational multivariate analysis of variance (ADONIS). Differences between LUIs among families were tested using *t*-test. The core microbiome was computed via QIIME and visualized with the Bioinformatics & Evolutionary Genomics webtool^1^. Ternary plots were constructed with the R package “vcd” using rarefied OTU tables. Constrained principal coordinate analyses were performed in R using the R package “vegan.” Further statistical analyses and visualization of the data were carried out in R using the packages “ggplot2,” “plyr,” “ade4,” and “ape.”

## Results

### Soil Characteristics

Water extractable organic carbon values were evenly distributed among different LUI levels. In contrast, WEON, nitrate, and ammonium concentrations changed with LUI levels. WEON and nitrate decreased from intensively to extensively managed sites. While WEON and nitrate levels were significantly higher in soil samples from the high LUI sites (Supplementary Table S1), ammonium concentrations were significantly higher under low land use (from 2.9–0.1 μg N g^-1^ dw). Plant carbon/nitrogen ratio increased from high to low land use intensity levels. The highest value (24.31 C/N) was detected on extensively managed sites.

### Characterization of Bacterial Diversity of Bulk Soil, Rhizosphere and Root Interior

A total of 3,883,552 raw reads were obtained. After quality filtering and chimera check with DeconSeq 3,853,784 remained, which could be clustered into a total of 3,241 OTUs at 97% sequence similarity. Afterward, plant derived 16S sequences (displayed by *Cyanobacteria* that were assigned to chloroplasts) were removed and output was filtered with an abundance cut-off of 0.005%, resulting in 2,903,916 reads. To make results comparable, the data set was rarefied to 11,052 reads per sample, reflecting the lowest obtained read number per sample. Rarefaction curves indicated that sampling depths were sufficient as plateaus were reached for samples from all compartments (Supplementary Figure S1).

**FIGURE 1:**
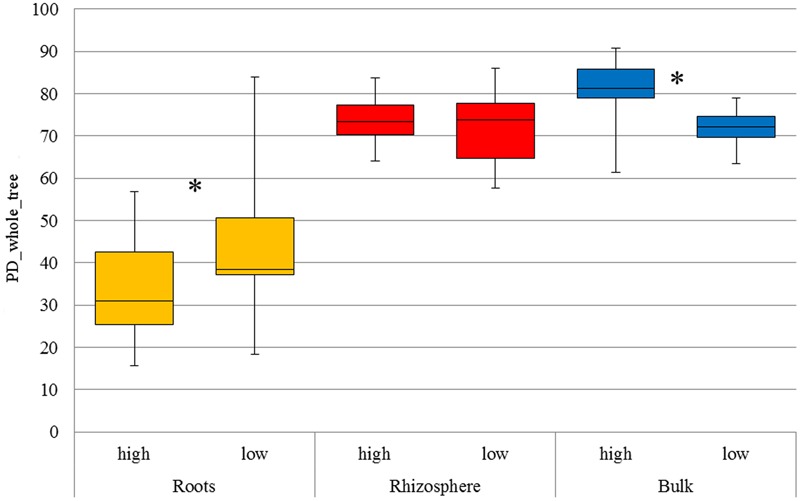
Faith’s phylogenetic diversity of all compartments under low and high land use intensity, respectively. The distribution of phylogenetic diversity is shown at 97% sequence similarity. The boxplot indicates the first and third quartile. The median is indicated as horizontal line and whiskers indicate minimum or maximum, respectively. Significant differences among land use intensities are indicated with an asterisk (*t*-test, *p* < 0.05).

As expected, α-diversity (**Figure 1**) was significantly lower in root samples (*p* < 0.05) compared to rhizosphere and bulk soil, respectively, for both LUIs. β-diversity indicated a clear separation of bacterial communities originating from root compared to rhizosphere and bulk soil, explaining up to 33% (weighted and unweighted Unifrac metrics) of the overall variance in the data (Supplementary Figure S2).

Phylogenetic classification of the obtained OTUs revealed 18 phyla, highlighting *Proteobacteria* as the dominating phylum in all compartments with increasing numbers from bulk soil (60%) and rhizosphere (69%) to roots (91%) (Supplementary Table S2A). In roots OTUs linked to *Pseudomonadaceae* were most abundant (15%). Other abundant OTUs in roots were linked to *Enterobacteriaceae* (12%), *Comamonadaceae* (10%), *Oxalobacteraceae* (9%), *Rhizobiaceae* (9%), *Sphingomonadaceae* (7%) and *Xanthomonadaceae* (6%). In contrast, in the rhizosphere, *Sinobacteriaceae* (9%), *Hyphomicrobiaceae* (8%), *Comamonadaceae* (7%), *Chitinophagaceae* (7%) and *Xanthomonadaceae* (6%) were found to be the most abundant families, while bulk soil was dominated by *Chitinophagaceae* (12%), *Hyphomicrobiaceae* (10%) and *Sinobacteriaceae* (9%) (Supplementary Table S2B). Besides *Proteobacteria, Bacteroidetes* (20/13/5%), *Actinobacteria* (9/10/2%) and *Acidobacteria* (4/4/1%) were also abundant in all compartments.

### The Influence of Land Use Intensity on Plant-Associated Bacterial Diversity

Analysis of α-diversity revealed a significant impact of LUI on bacterial diversity in bulk soil as well as in roots (*p* < 0.05), with higher α-diversity values in bulk soil and lower α-diversity in the root interior under high LUI (**Figure 1**). Effects on the microbiome diversity in the rhizosphere in response to LUI were not significant (*p* > 0.05). Furthermore, significant changes in β-diversity in response to land use intensity were revealed by permutational multivariate analysis of variance (ADONIS) using qualitative measures (unweighted Unifrac distance matrix) for all three compartments. In contrast, no significant difference was detected on Bray–Curtis dissimilarity or weighted Unifrac distance in roots and rhizosphere, leading to the assumption that significance is a result of presence and/or absence of certain OTUs. However, in bulk soil LUI also significantly affected community composition in relative abundance (Bray–Curtis; Supplementary Table S3).

**FIGURE 2:**
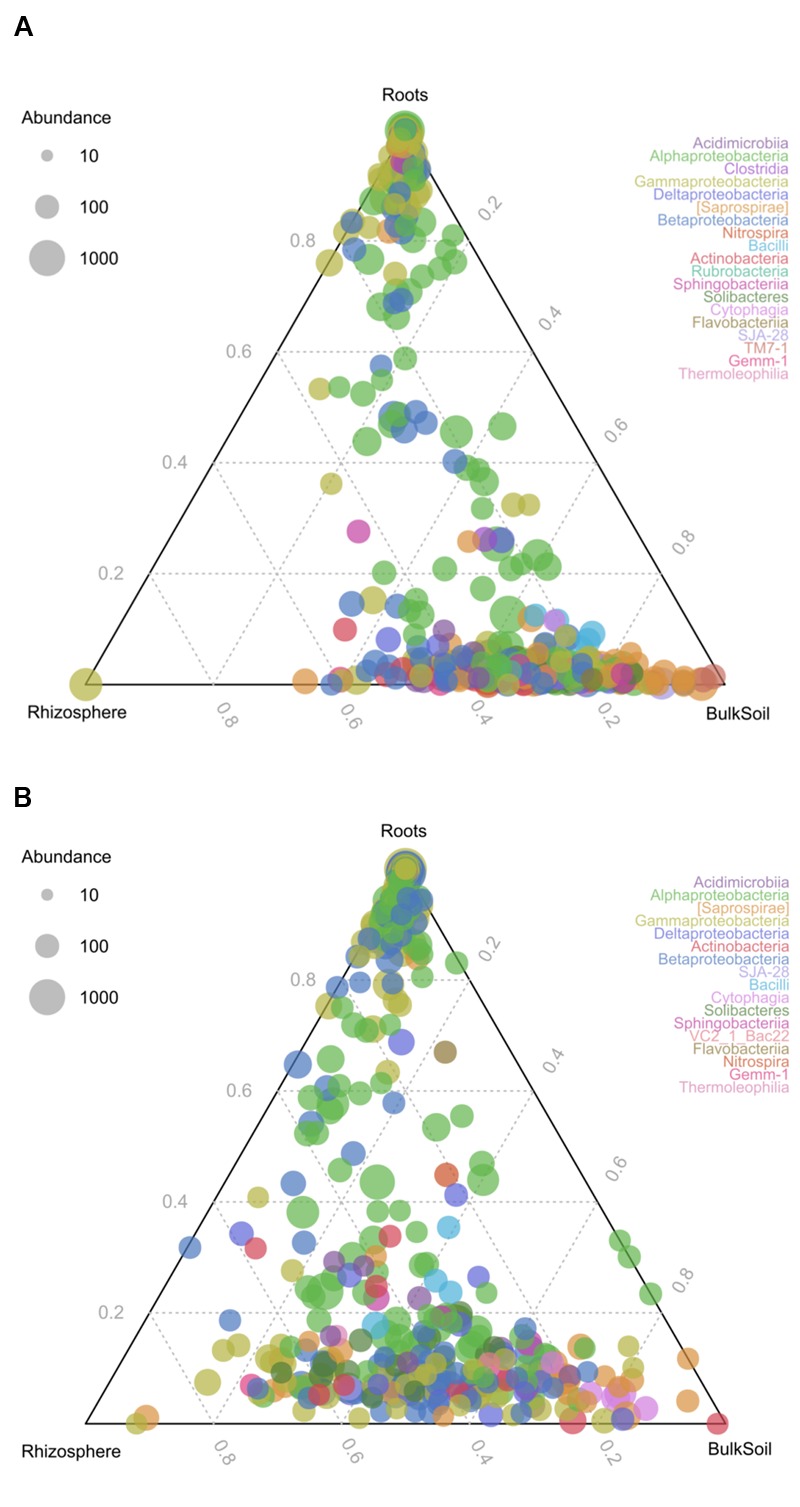
Ternary plots of community structure in roots, rhizosphere and bulk soil. The colors represent the classes to which corresponding OTUs were assigned. **(A)** Represents distribution under high LUI, **(B)** under low LUI. The size of the plotted dots corresponds to the abundance of the OTUs with respect to each compartment. The position of each circle is determined by the contribution of the indicated compartments to the total abundance.

To identify changes in bacterial communities as a consequence of different LUIs in the three compartments, ternary plots were prepared (**Figure 2**). The results reveal that roots harbored distinct communities under both LUI. However, a shift in community structure can be observed as a consequence of land use. At high LUI, a large number of OTUs was almost exclusively shared between rhizosphere and bulk soil and was not found in the root interior. At low LUI a remarkable amount of OTUs could be found in the root interior and in the rhizosphere suggesting a specific recruitment of bacterial taxa by the plant.

To gain deeper insights into the shared communities in all compartments (OTUs present across 95% of the samples) under low or high LUI, respectively, an analysis of the core microbiome was performed. Consistent with the ternary plot, more shared OTUs were found under low LUI (38 OTUs) compared to high LUI (17 OTUs). Among these, a shared core of 12 OTUs could be defined which was independent of compartment and LUI (Supplementary Figure S3). These OTUs were assigned to *Bradyrhizobiaceae* (4 OTUs), *Sphingomonadaceae* (2 OTUs), *Pseudomonadaceae* (1 OTUs), *Comamonadaceae* (1 OTU), *Hyphomicrobiaceae* (2 OTUs), *Caulobacteraceae* (1 OTUs), and *Rhizobiaceae* (1 OTU). OTUs shared under low or high LUI are presented in Supplementary Tables S4–S6.

Significant differences with respect to LUI in the abundance of assigned families in roots could only be detected for four taxa (Supplementary Table S7A). Two families assigned to *Bryobacteraceae* and *Cytophagaceae* and unassigned members of *Rhizobiales* were more present under low LUI while one taxon was more prominent under high LUI (*Turicibacteraceae*). In contrast, 19 families were found to significantly differ among LUIs in the rhizosphere (Supplementary Table S7B) and 48 in bulk soil, respectively (Supplementary Table S7C; *p*-value < 0.05). Interestingly, only two of the taxa that were significantly affected by LUI in roots appeared to be also affected in the other compartments. *Turicibacteraceae* were influenced in bulk soil as well as unassigned members of *Rhizobiales* in rhizosphere (*p* < 0.05). However, LUI did not have a significant impact on the most abundant families colonizing the root interior.

**FIGURE 3:**
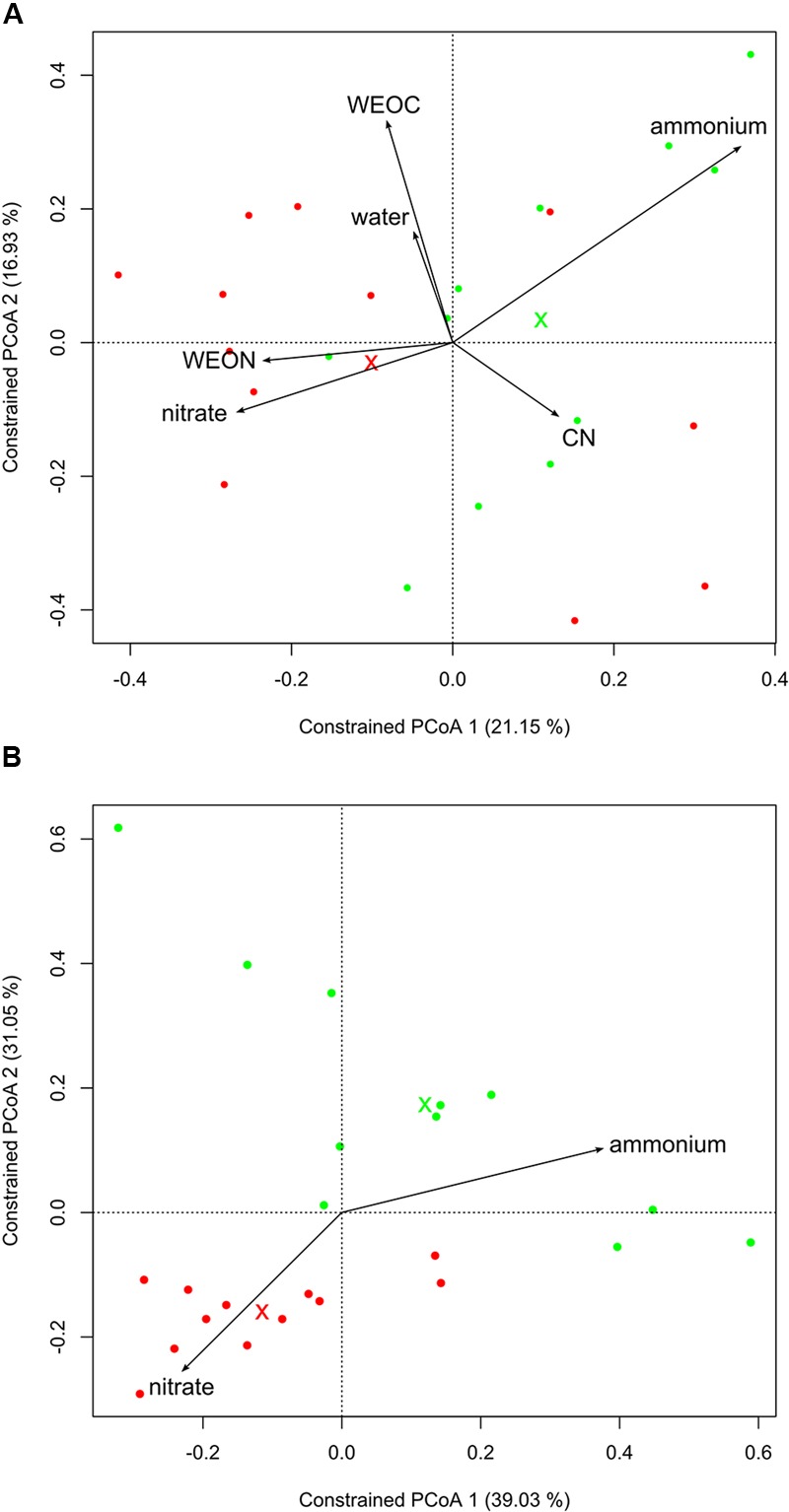
Canonical analysis of principal coordinates of Bray–Curtis dissimilarities in roots. Crosses mark centroids of low or high LUI, respectively. Low LUI is represented in green, high LUI is represented in red color. **(A)** Using soil characteristics as constraints. Constraints reflect 36% of overall variance in the data (PERMANOVA, *p* < 0.003; 95% confidence interval), **(B)** using soil nitrate and ammonium as constrains independent from other soil characteristics. Constraints reflect 16% of overall variance in the data (PERMANOVA, *p* < 0.019; 95% confidence interval).

To better understand the driving factors of the differences in bacterial community structure in the three compartments, we performed a canonical analysis of principal coordinates. Including the soil characteristics in the ordination revealed that LUI is best represented by nitrate (high LUI) and ammonium (low LUI). The soil characteristics used explained 36% of the variation of microbial community structure in the roots (**Figure 3A**). Apart from compartment, LUI and WEON appeared to be the main driving forces for bacterial community composition in the endosphere and rhizosphere, whereas in bulk soil, all characteristics were strong determinants of community structure. Using nitrate and ammonium as constraints while excluding the other parameters explained 16% of the variation in bacterial community structure of the root endosphere (**Figure 3B**, PERMANOVA; *p* < 0.05). In the rhizosphere, 20% could be explained and in bulk soil 28% of variation in microbial community structure could be explained by soil ammonium and nitrogen (PERMANOVA; *p* < 0.001).

## Discussion

### Microbial Diversity in Different Soil and Plant Compartments

The phylogenetic classification of plant-associated bacterial communities of *D. glomerata* highlighted *Proteobacteria* as the dominant phylum in all compartments, whereas other phyla including *Acidobacteria* and *Actinobacteria* were less present. *Proteobacteria* were found to be the predominating phylum across several plant species, including *Arabidopsis, Lotus japonicus, Hordeum vulgare* and *Agave* ssp. (Bulgarelli et al., 2012, 2015; Coleman-Derr et al., 2016; Zgadzaj et al., 2016). However, our data are only partly consistent with other studies performed in the same area. Kaiser et al. (2016) reported that beside *Proteobacteria, Acidobacteria* and *Actinobacteria* are highly abundant in soils from the “Schwäbische Alb.” Similar results were obtained in other studies as well (Foesel et al., 2014). However, in both studies samplings were performed during spring, whereas our sampling took place in early summer. Accordingly, this change might be due to seasonal shifts in resource availability, which is indirectly caused by the plant composition through C allocation or nutrient uptake (Koranda et al., 2013). Furthermore, as the microbiome in bulk soil appeared to be fairly similar to rhizosphere, it is likely that the sampled “bulk soil” in our study has been strongly influenced by the surrounding plants.

Moreover, our results revealed that *Pseudomonadaceae* are highly abundant in the root endosphere, which is in line with other studies investigating plant colonizing bacteria (Wemheuer et al., 2017). *Pseudomonadaceae* are reported to exhibit a wide range of plant-beneficial properties and plant growth-promoting traits. These include phosphate solubilization, nitrogen fixation, synthesis of cytokinins as well as of the phytohormones indole-3-acetic acid (IAA) and gibberellins (Cabanas et al., 2014; Vacheron et al., 2016). The latter was reported to affect seed formation and ripening in grasses (Stoddart, 1965; Kim et al., 2016), emphasizing the importance of *Pseudomonadaceae* in our study. The other abundant phyla detected in roots were *Rhizobiaceae*. Microbes of this family are known for antifungal properties as well as for plant growth-promoting, phosphate solubilizing and nitrogen-fixing abilities (Haack et al., 2016). Several members of *Oxalobacteraceae*, as well as *Enterobacteriaceae* and *Comamonadaceae* are frequently reported to be found inside roots and to possess plant-growth promoting effects (Bulgarelli et al., 2013; Taghavi et al., 2015; Cope-Selby et al., 2017). In contrast to our study, Wemheuer et al. (2017) described *Massilia*, which is a genus of *Oxalobacteraceae*, as the most abundant root endophyte in *D. glomerata*. Even though *Oxalobacteraceae* were highly abundant root endophytes in our study, we could not detect the genus *Massilia* within our samples. To examine this in detail with careful and critical attention, further studies on the genus level have to be performed. Whereas Wemheuer et al. (2017) took their samples in autumn, when plants have already reached a senescence state, in our study samples were obtained during early summer, in the reproductive phase of the plant. Various studies have shown that both, land use and sampling season, influence the plant phenotype which in turn changes microbial patterns (Weaver, 1946; Bevivino et al., 2014). While this might explain the difference of abundant OTUs in both studies, another possible explanation for this incongruence might be the influence of various parameters like grazing, soil type, texture and chemical properties on bacterial community composition. Indeed, the latter appears to be highly probable since this genus was not found in other studies conducted at the same site (Kaiser et al., 2016).

### Influence of Land Use Intensity on Plant-Associated Microbiomes

In this study, the main aim was to identify the impact of LUI on the plant-associated microbiome of *D. glomerata* during its reproductive stage. Interestingly, an increase of α-diversity was observed under intensive land use in bulk soil, which is in contrast to our initial hypothesis. This might be explained by an uneven distribution of certain bacterial taxa under high LUI, as it has been postulated that composition of microbial communities in intensively managed habitats tend to be highly variable (Garbeva et al., 2008). This in turn might lead to a transient and patchy colonization of the roots (Gottel et al., 2011). In contrast, microbial community composition in natural habitats with low human impact is considered to be more stable and thus colonization is more uniform (Bevivino et al., 2014). In this study, there was no influence of LUI on the microbial diversity in the rhizosphere. This corresponds to numerous studies that showed that rhizosphere microbial communities are more strongly affected by plant species rather than soil characteristics (Grayston et al., 1998; Gottel et al., 2011). However, a significant effect of LUI on the diversity was found in the roots. Analysis of differences in microbial communities revealed significant changes in qualitative measures (unweighted Unifrac) between high and low LUI in all compartments. These findings indicate that the divergence of the root and rhizosphere colonizing microbiota originates from the presence/absence of single OTUs across different LUIs, rather than on differences in the abundances of OTUs from different phyla. Significance in roots is therefore attributed to low-abundance OTUs, which are present at sites under low LUI or high LUI, respectively. However, most research has focused on the dominant community members rather than on less abundant phyla in the past. Still, abundance is not intimately connected with high metabolic activity (Hunt et al., 2013). Instead, the less abundant phylotypes might be metabolically more active or specific, thus strongly influencing the functional potential of microbial communities (Jousset et al., 2017). Hence, further investigations on the activity of microbial communities and their metabolic activities are required.

Nevertheless, results of diversity analysis corroborate our findings on the significant differences of the abundant bacterial families in roots. Most abundant taxa in the endosphere were not affected by LUI. It has been proposed that host tissues represent unique niches for associated bacteria, leading to little variation in dominant communities (Gottel et al., 2011). Only four families were significantly affected by LUI: *Turicibacteraceae, Bryobacteraceae, Cytophagaceae* and unassigned members of the *Rhizobiales*, albeit all of them were found in very low abundances (each less than 1% across all samples). Numerous families within *Rhizobiales*, e.g., *Rhizobiaceae*, are known for their plant-growth promoting and nitrogen-fixing abilities in *Fabaceae* (Patriarca et al., 2002; Chi et al., 2005). However, members of *Rhizobiales* were also found to express *nifH* in roots of sugarcane, a gene which encodes enzymes involved in biological nitrogen fixation (Rouws et al., 2014), thus enhancing plant performance under limited nitrogen availability. These findings indicate that *Rhizobiales* also play an important role in perennial grasses, albeit without nodule formation. Added to this, families of *Bryobacteraceae* were characterized as chemoheterotrophs that utilize various sugars and polysaccharides (Dedysh et al., 2016) and strains of *Cytophagaceae* are often reported to be able to fix nitrogen, digest polysaccharides or proteins as well as to utilize cellulose, making these (especially under low LUI) important for recycling of the most abundant carbohydrates produced by plants. Species belonging to these families have already been isolated from rhizosphere as well as plant tissues (McBride et al., 2014; Xu et al., 2014). In addition, *Turicibacteraceae* were mostly found across high LUIs. Species of this family are commonly found in the guts of animals (Auchtung et al., 2016), which could have been transferred through fertilization with manure or grazing by cattle in intensively managed sites. As a consequence, they were not further characterized as a plant growth-promoting taxon in other studies. However, *Turicibacteraceae* apparently are able to enter the endosphere of *D. glomerata* without causing visible disease symptoms. The frequency of these less abundant taxa suggests that they are sporadically acquired from the immediate environment, since they were not present in the core microbiome (Santhanam et al., 2015).

Together with the analysis of microbial patterns between the compartments (**Figure 2**), the results of the present study suggest the existence of a distinct microbial community in roots compared to rhizosphere and soil under both LUIs. Interestingly, a strong shift of community structure was observed from high to low LUI, leading to remarkably higher amounts of shared OTUs in samples from sites under low LUIs. Among OTUs uniquely found to be shared in all compartments under low LUI are OTUs which show close similarity to *Mesorhizobium*, which is well-known for its nitrogen-fixing and plant growth-promoting functions (Laranjo et al., 2014); this suggests that the plant recruits its microbiome from its surrounding soil in response to lower nutrient availability. Interaction between the plant and soil therefore seems to be more pronounced and specific under extensive land use.

Previous studies had indicated the strong influence of plants on the microbial colonization in the rhizosphere through exudation. However, it remained unclear whether soil type or selection by the host plant was the major determinant of root microbiome composition (Girvan et al., 2003; Berg and Smalla, 2009; Berg et al., 2014). Since the soil type was identical in the plots of our study, compartments revealed 26% of variation (Bray–Curtis) in the data, whereas the soil properties nitrate and ammonium together explained only 16% of overall variation in roots. Based on these results we suggest an indirect contribution of nitrogen availability on the colonization of microbial communities in the plant. Recent studies have suggested that plants are able to sense their external and internal nitrogen status, demonstrating their ability to monitor and respond to changes to external nitrogen as well as endogenous nitrogen availability (Gent and Forde, 2017). Under low LUI, less nitrogen is available for the plant (see C/N ratio and soil characteristics in Supplementary Table S1 and Figure S1). Thus, the plants need to adjust to this persisting lower nitrogen status, which in turn might lead to change in exudation to influence its surrounding environment to their benefit (Lareen et al., 2016). In contrast, under high land use intensity, a high amount of nitrogen is introduced through fertilization, making specific attraction less important. Hence, land use intensity has an influence on the plant-specific traits that shape the plant–soil interface and thus indirectly influences the root microbial community.

## Conclusion and Outlook

Our results indicate that roots of reproducing *D. glomerata* plants exhibit a unique niche for associated bacteria, which leads to a distinctive microbiome independent of land use intensity. Only rare root inhabiting taxa were influenced by LUI. We assume an indirect influence of LUI on the plant-associated microbiome as we observed a strong shift of community structure on the plant–soil interface. However, as abundance might not directly inform on metabolic activity, further more process oriented investigations are required addressing the functional potential of those microbial communities. Additionally, analysis of microbial composition in roots of *D. glomerata* in different plant developmental stages as well as root exudation may enhance knowledge on the dynamics of establishment of specific microbiomes at the plant–soil interface in response to land use intensity.

## Author Contributions

JE designed the experiment, carried out the field work, laboratory experiments, data analysis and wrote the manuscript. BS and PS contributed to the design of the experiment. PH contributed to the field work and the laboratory experiments. GV provided the pipelines for sequencing data analysis and advised data analysis. BS, PS, GV, JJ, MR, and MS advised the studies and critically revised the manuscript.

## Conflict of Interest Statement

The authors declare that the research was conducted in the absence of any commercial or financial relationships that could be construed as a potential conflict of interest.

## Footnotes

1 http://bioinformatics.psb.ugent.be/webtools/Venn/
