# Supplementary material for: The Influence of Land Use Intensity on the Plant-Associated Microbiome of Dactylis glomerata L
Source: Front Plant Sci. 2017 Jun 21;8:930. doi: 10.3389/fpls.2017.00930 (PMC5478725; doi:10.3389/fpls.2017.00930)
Supplement: Supplementary file 1 [file Data_Sheet_1.doc]

Supplementary Material

The Influence of Land Use Intensity on the Plant Associated Microbiome of *Dactylis glomerata* L.

**Jennifer Estendorfer, Barbara Stempfhuber, Paula Haury, Gisle Vestergaard, Matthias C. Rillig, Jasmin Joshi, Peter Schröder*, Michael Schloter**

*** Correspondence:** Peter Schröder: [peter.schroeder@helmholtz-muenchen.de](mailto:peter.schroeder@helmholtz-muenchen.de)

# Supplementary Figures and Tables

## Supplementary Figures

**Figure S1: Rarefaction curve of phylogenetic diversity in all compartments under low and high LUI at 97% sequence similarity.** Bulk soil is depicted in blue, rhizosphere in red and root endosphere in orange. Lighter colors indicate low LUI.

**Figure S2: Principal coordinate analysis (PCoA) based on dissimilarity matrices (95% confidence intervals).** **(A)** unweighted Unifrac (R^2^= 0.33, p<0.001), **(B)** weighted Unifrac (R^2^= 0.33, p<0.001), **(C)** Bray-Curtis (R^2^= 0.26, p<0.001)**.**

**Figure S3: VennDiagram of shared OTUs between all compartments in high versus low LUI.** Red circle depicts high LUI, green circle depicts low LUI. Percentages refer to the total data.

## Supplementary Tables

**Table S1: Summary of edaphic parameters.** Water extractable carbon/nitrogen (WEOC/WEON), nitrate, ammonium, C/N ratio in plants, gravimetric water content in the soil (water), Index: land use intensity index, calculated for 2006-2014, as described in Blüthgen et al. (2012) for grassland samples, the management and treatment of sampled sites.

**Table S2: Relative abundance of assigned taxa.** Groups that were less abundant than 1% in all compartments were grouped together in “Others”. (A) most abundant phyla (B) most abundant families

**Table S3: The impact of LUI on the β-diversity (between sample diversity) of bacterial community compositions.** Significance (ADONIS, p-value < 0.05) indicated in bold letters, marginal significance (ADONIS, p-value < 0.1) indicated in italics.

**Table S4: Classification of shared core OTUs that were found in 95% of all samples.**

**Table S5: Classification of core OTUs that were found in 95% of all samples only under low land use intensities.**

**Table S6: Classification of core OTUs that were found in 95% of all samples only under high land use intensities.**

**Table S7: Families that are significantly affected by land use intensity.** Significance was tested using t-test. Marginal significance (p <0.1) is indicated in italics **(A)** in roots, **(B)** in rhizosphere and **(C)** in bulk soil

**Figure S1**


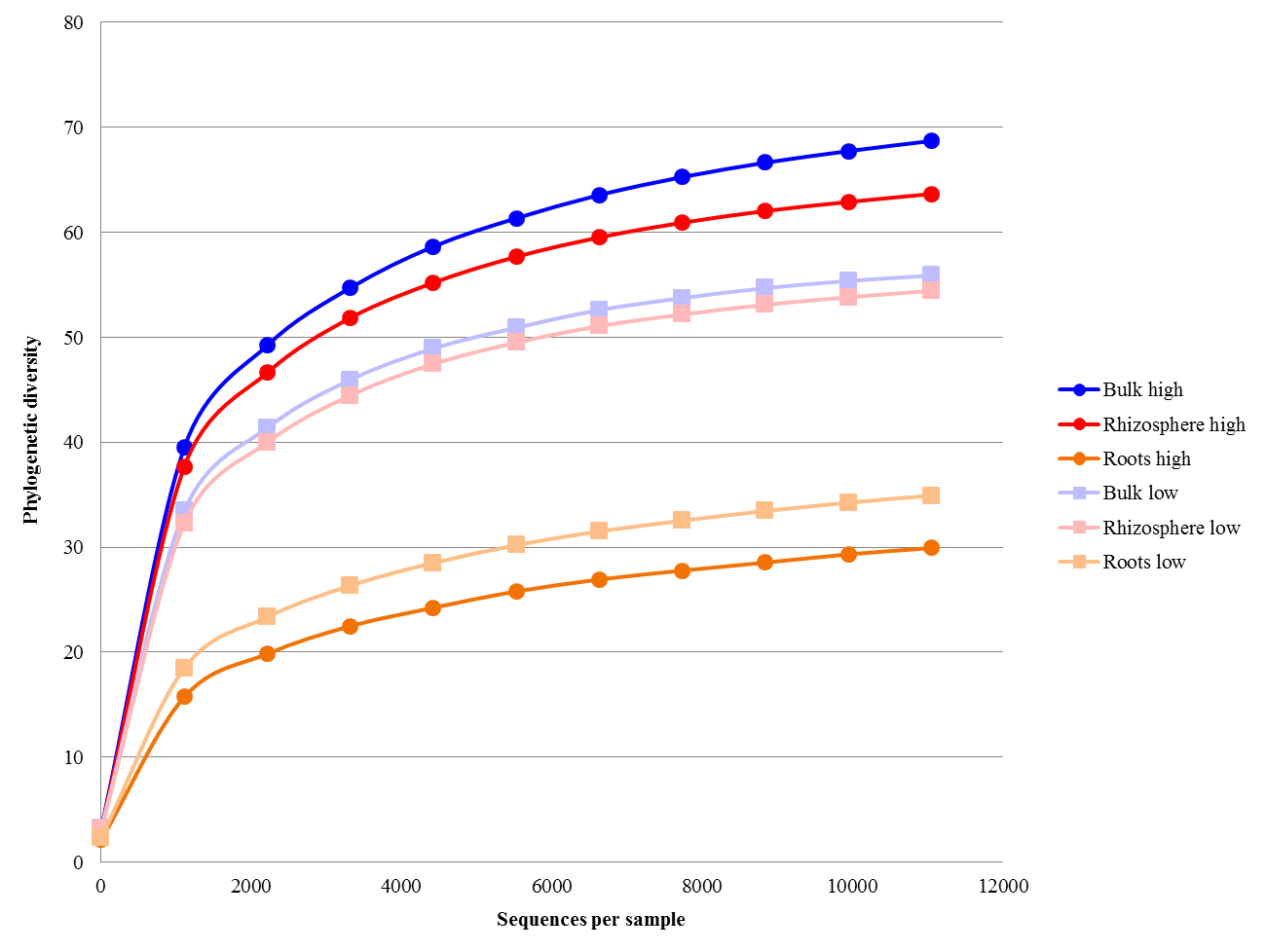


**Figure S2A**


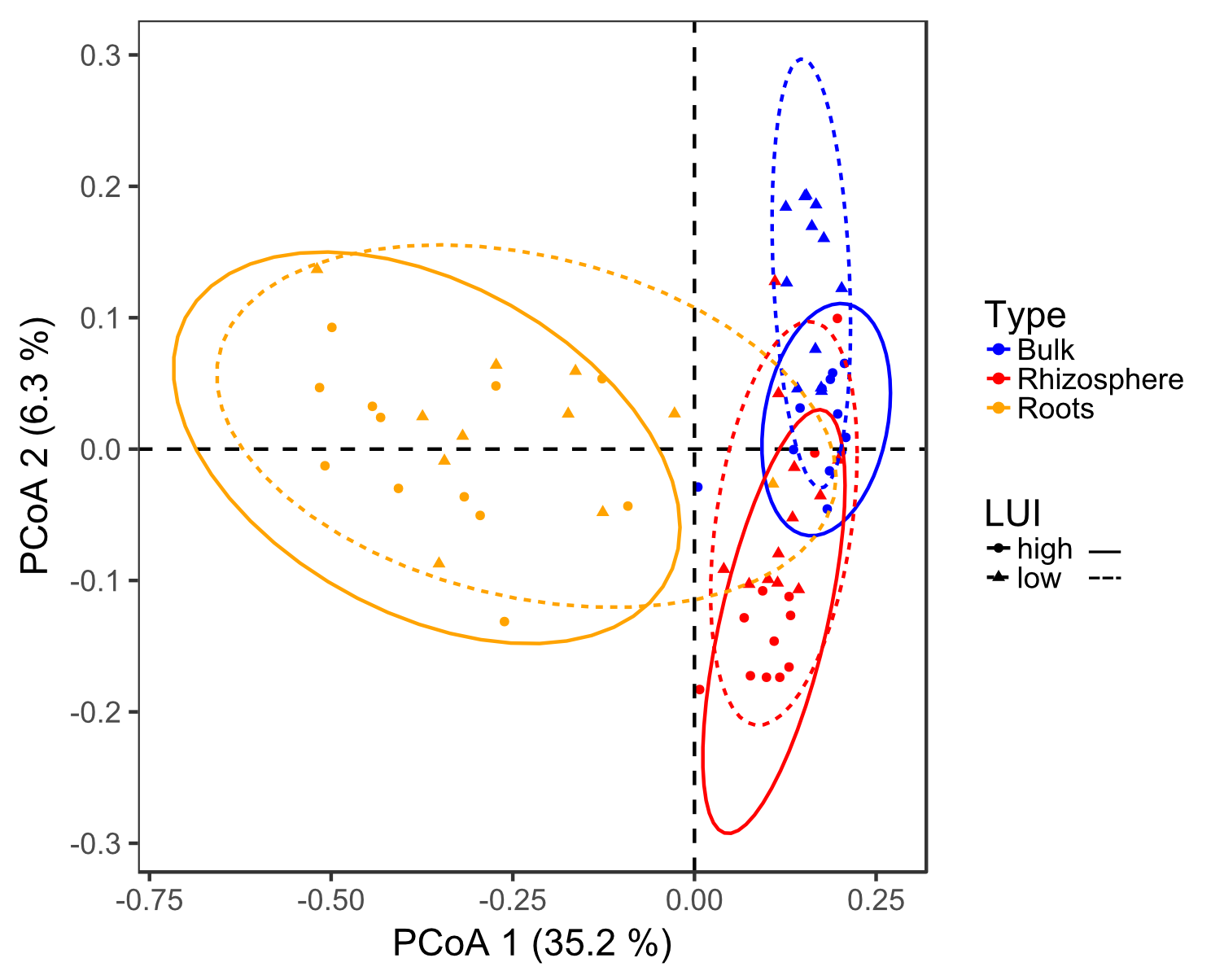


**Figure 2B**


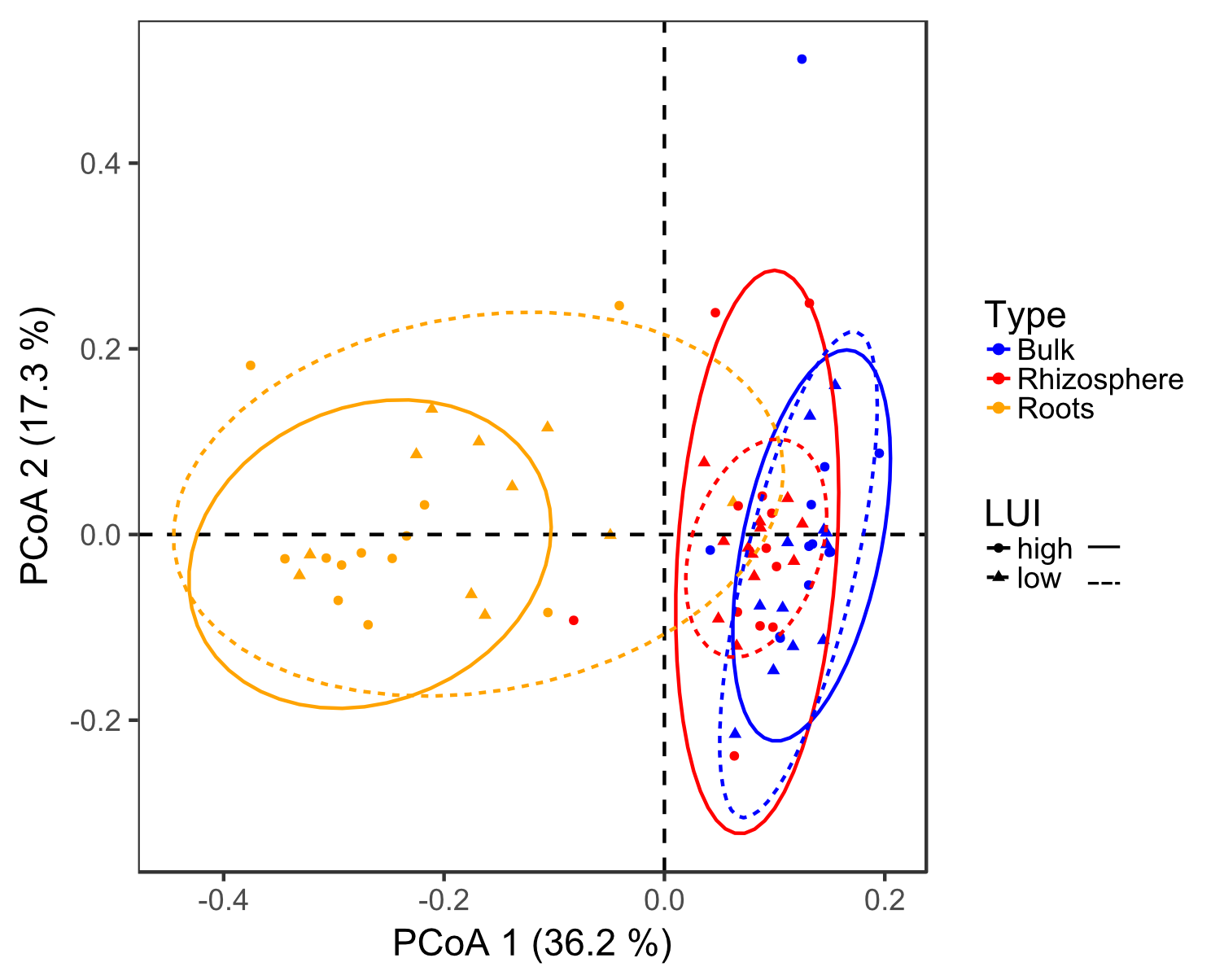


**Figure 2C**


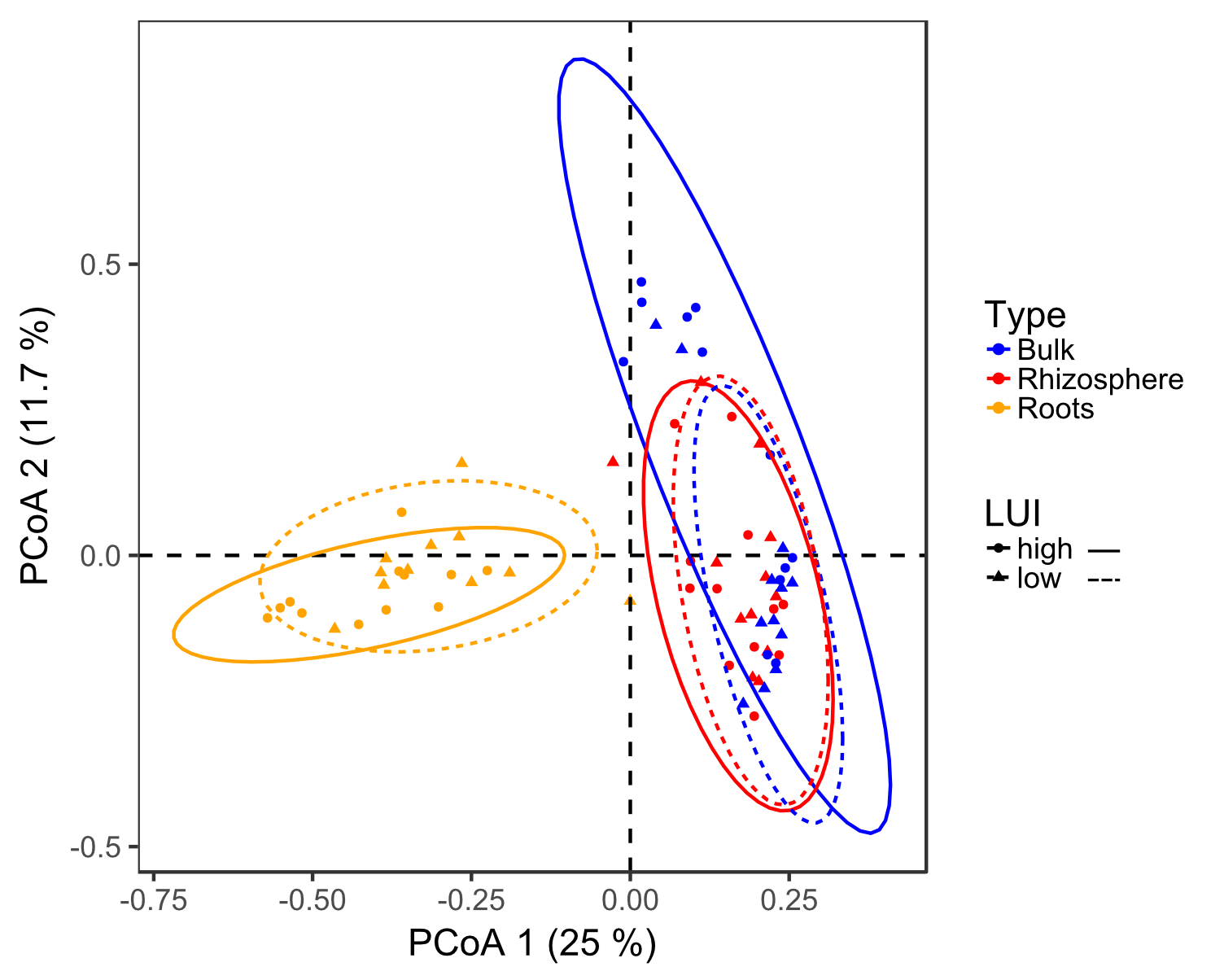


**Figure S3**

12

26

16.2%

5

8.4%

high

low

Table S1

| **Plot ID** | **WEOC [µg g-1 dw]** | **WEON [µg g-1 dw]** | **Nitrate [µg N g-1 dw]** | **Ammonium [µg N g-1 dw]** | **C/N ratio plants** | **Water (%)** | **Index (2006-2014)** | **Management** | **Treatment** |
| --- | --- | --- | --- | --- | --- | --- | --- | --- | --- |
| **AEG6** | 25.84±5.55 | 40.65±8.23 | 42.29±4.53 | 0.1±0.04 | 19.73±2.78 | 37 | 2.25 | mown pasture | fertilized |
| **AEG19** | 30.25±7.8 | 41.87±9.41 | 45.57±9.13 | 0.11±0.03 | 12.25±0.19 | 34 | 2.28 | mown pasture | fertilized |
| **AEG20** | 24.75±4.76 | 48.42±11.92 | 51.04±7.26 | 0.11±0.02 | 13.04±1.41 | 44 | 1.55 | pasture | fertilized |
| **AEG21** | 25.56±4.5 | 41.57±12.2 | 45.43±11.04 | 0.13±0.06 | 14.51±1.39 | 36 | 3.62 | pasture | fertilized |
| **AEG7** | 27.54±21.35 | 25.61±5.79 | 17.03±4.87 | 5.76±1.57 | 16.65±0.21 | 40 | 0.56 | pasture | non-fertilized |
| **AEG28** | 25.08±6.02 | 14.46±3.8 | 12.11±2.93 | 2.28±0.49 | 25.78±1.85 | 34 | 1.06 | pasture | non-fertilized |
| **AEG33** | 25.48±8.08 | 8.67±1.56 | 4.55±2.48 | 2.81±0.91 | 26.46±1.27 | 31 | 1.32 | pasture | non-fertilized |
| **AEG34** | 24.05±4.71 | 21.25±4.59 | 24.77±5.19 | 0.79±0.38 | 28.33±0.28 | 30 | 1.31 | pasture | non-fertilized |

Table S2A

| **Phylum** | **Bulk RA (%)** | **Rhizosphere RA (%)** | **Roots RA (%)** |
| --- | --- | --- | --- |
| Proteobacteria | 0.60 | 0.69 | 0.91 |
| Bacteroidetes | 0.20 | 0.13 | 0.05 |
| Actinobacteria | 0.09 | 0.10 | 0.02 |
| Acidobacteria | 0.04 | 0.04 | 0.01 |
| Firmicutes | 0.02 | 0.01 | 0.01 |
| Nitrospirae | 0.01 | 0.01 | <0.01 |
| TM7 | 0.01 | <0.01 | <0.01 |
| Chlorobi | 0.01 | <0.01 | <0.01 |
| Gemmatimonadetes | 0.01 | 0.01 | <0.01 |
| Others | 0.01 | <0.01 | <0.01 |

Table S2B

| **Family** | **Bulk RA (%)** | **Rhizosphere RA (%)** | **Roots RA (%)** |
| --- | --- | --- | --- |
| Pseudomonadaceae | 0.01 | 0.01 | 0.15 |
| Enterobacteriaceae | <0.01 | 0.01 | 0.12 |
| Comamonadaceae | 0.04 | 0.07 | 0.10 |
| Oxalobacteraceae | <0.01 | 0.01 | 0.09 |
| Rhizobiaceae | <0.01 | 0.01 | 0.09 |
| Sphingomonadaceae | 0.02 | 0.03 | 0.07 |
| Xanthomonadaceae | 0.03 | 0.06 | 0.06 |
| Bradyrhizobiaceae | 0.04 | 0.05 | 0.05 |
| Sinobacteraceae | 0.09 | 0.09 | 0.04 |
| Burkholderiaceae | <0.01 | <0.01 | 0.03 |
| Phyllobacteriaceae | 0.01 | 0.01 | 0.02 |
| Chitinophagaceae | 0.12 | 0.07 | 0.02 |
| Hyphomicrobiaceae | 0.10 | 0.08 | 0.02 |
| Sphingobacteriaceae | <0.01 | 0.01 | 0.02 |
| Caulobacteraceae | <0.01 | 0.01 | 0.02 |
| Rhodospirillaceae | 0.02 | 0.02 | 0.01 |
| Xanthobacteraceae | <0.01 | <0.01 | 0.01 |
| Haliangiaceae | 0.02 | 0.01 | 0.01 |
| Mycobacteriaceae | <0.01 | 0.01 | <0.01 |
| Flavobacteriaceae | 0.01 | 0.01 | <0.01 |
| Solibacteraceae | 0.02 | 0.02 | <0.01 |
| Propionibacteriaceae | 0.01 | 0.01 | <0.01 |
| Cytophagaceae | 0.02 | 0.01 | <0.01 |
| Bacillaceae | 0.01 | 0.01 | <0.01 |
| Micromonosporaceae | <0.01 | 0.01 | <0.01 |
| C111 | 0.01 | 0.01 | <0.01 |
| Alcaligenaceae | 0.01 | 0.01 | <0.01 |
| EB1017 | 0.01 | 0.01 | <0.01 |
| Nitrospiraceae | 0.01 | 0.01 | <0.01 |
| Gaiellaceae | 0.01 | 0.01 | <0.01 |
| Syntrophobacteraceae | 0.01 | 0.01 | <0.01 |
| Saprospiraceae | 0.01 | 0.01 | <0.01 |
| Piscirickettsiaceae | 0.01 | <0.01 | <0.01 |
| Others | 0.32 | 0.31 | 0.06 |

Table S3

| Compartment | Distance metrics | R^2^ | p-value |
| --- | --- | --- | --- |
| Roots | **Unweighted** | **0.08093** | **0.015** |
|  | Weighted | 0.05146 | 0.278 |
|  | Bray-Curtis | 0.05291 | 0.16 |
| Rhizosphere | **Unweighted** | **0.08949** | **0.003** |
|  | Weighted | 0.05793 | 0.202 |
|  | *Bray-Curtis* | *0.07037* | *0.068* |
| Bulk soil | **Unweighted** | **0.1441** | **0.001** |
|  | Weighted | 0.0747 | 0.124 |
|  | **Bray-Curtis** | **0.13575** | **0.008** |

Table S4

| **OTU ID** | **Kingdom** | **Phylum** | **Class** | **Order** | **Family** | **Genus** |
| --- | --- | --- | --- | --- | --- | --- |
| **573764** | Bacteria | Proteobacteria | Alphaproteobacteria | Rhizobiales | Bradyrhizobiaceae | *Bradyrhizobium* |
| **4377104** | Bacteria | Proteobacteria | Alphaproteobacteria | Rhizobiales | Bradyrhizobiaceae | *Bradyrhizobium* |
| **1105814** | Bacteria | Proteobacteria | Alphaproteobacteria | Rhizobiales | Bradyrhizobiaceae | *Bradyrhizobium* |
| **580625** | Bacteria | Proteobacteria | Alphaproteobacteria | Rhizobiales | Bradyrhizobiaceae | *Bradyrhizobium* |
| **866365** | Bacteria | Proteobacteria | Alphaproteobacteria | Caulobacterales | Caulobacteraceae | *Caulobacter* |
| **731707** | Bacteria | Proteobacteria | Betaproteobacteria | Burkholderiales | Comamonadaceae | *Variovorax* |
| **317632** | Bacteria | Proteobacteria | Alphaproteobacteria | Rhizobiales | Hyphomicrobiaceae | *Rhodoplanes* |
| **573013** | Bacteria | Proteobacteria | Alphaproteobacteria | Rhizobiales | Hyphomicrobiaceae | *Devosia* |
| **646549** | Bacteria | Proteobacteria | Gammaproteobacteria | Pseudomonadales | Pseudomonadaceae | *Pseudomonas* |
| **969805** | Bacteria | Proteobacteria | Alphaproteobacteria | Rhizobiales | Rhizobiaceae | *Agrobacterium* |
| **1003206** | Bacteria | Proteobacteria | Alphaproteobacteria | Sphingomonadales | Sphingomonadaceae | *Sphingomonas* |
| **573258** | Bacteria | Proteobacteria | Alphaproteobacteria | Sphingomonadales | Sphingomonadaceae | *Kaistobacter* |

Table S5

| **OTU ID** | **Kingdom** | **Phylum** | **Class** | **Order** | **Family** | **Genus** |
| --- | --- | --- | --- | --- | --- | --- |
| **399818** | Bacteria | Proteobacteria | Betaproteobacteria | Ellin6067 | Not assigned | Not assigned |
| **113261** | Bacteria | Proteobacteria | Deltaproteobacteria | Myxococcales | Not assigned | Not assigned |
| **254098** | Bacteria | Proteobacteria | Betaproteobacteria | Not assigned | Not assigned | Not assigned |
| **838594** | Bacteria | Firmicutes | Bacilli | Bacillales | Bacillaceae | *Bacillus* |
| **961922** | Bacteria | Firmicutes | Bacilli | Bacillales | Bacillaceae | *Bacillus* |
| **580703** | Bacteria | Firmicutes | Bacilli | Bacillales | Bacillaceae | *Bacillus* |
| **573135** | Bacteria | Proteobacteria | Alphaproteobacteria | Rhizobiales | Bradyrhizobiaceae | *Bradyrhizobium* |
| **358785** | Bacteria | Proteobacteria | Alphaproteobacteria | Rhizobiales | Hyphomicrobiaceae | *Rhodoplanes* |
| **545247** | Bacteria | Proteobacteria | Alphaproteobacteria | Rhizobiales | Hyphomicrobiaceae | *Rhodoplanes* |
| **616682** | Bacteria | Proteobacteria | Alphaproteobacteria | Rhizobiales | Hyphomicrobiaceae | *Rhodoplanes* |
| **2025156** | Bacteria | Proteobacteria | Alphaproteobacteria | Rhizobiales | Hyphomicrobiaceae | *Rhodoplanes* |
| **210914** | Bacteria | Proteobacteria | Alphaproteobacteria | Rhizobiales | Hyphomicrobiaceae | *Rhodoplanes* |
| **New.0.CleanUp.ReferenceOTU4459** | Bacteria | Proteobacteria | Alphaproteobacteria | Rhizobiales | Hyphomicrobiaceae | *Not assigned* |
| **New.1.CleanUp.ReferenceOTU99** | Bacteria | Proteobacteria | Alphaproteobacteria | Rhizobiales | Hyphomicrobiaceae | *Rhodoplanes* |
| **620684** | Bacteria | Proteobacteria | Alphaproteobacteria | Rhizobiales | Phyllobacteriaceae | *Mesorhizobium* |
| **547097** | Bacteria | Proteobacteria | Alphaproteobacteria | Rhizobiales | Phyllobacteriaceae | *Mesorhizobium* |
| **571263** | Bacteria | Proteobacteria | Alphaproteobacteria | Rhizobiales | Phyllobacteriaceae | *Mesorhizobium* |
| **398604** | Bacteria | Proteobacteria | Gammaproteobacteria | Pseudomonadales | Pseudomonadaceae | *Pseudomonas* |
| **350105** | Bacteria | Proteobacteria | Gammaproteobacteria | Pseudomonadales | Pseudomonadaceae | *Pseudomonas* |
| **3314521** | Bacteria | Proteobacteria | Gammaproteobacteria | Pseudomonadales | Pseudomonadaceae | *Pseudomonas* |
| **1104627** | Bacteria | Proteobacteria | Alphaproteobacteria | Rhizobiales | Rhizobiaceae | *Rhizobium* |
| **709657** | Bacteria | Proteobacteria | Alphaproteobacteria | Rhodospirillales | Rhodospirillaceae | Not assigned |
| **281360** | Bacteria | Proteobacteria | Alphaproteobacteria | Rhodospirillales | Rhodospirillaceae | Not assigned |
| **4373617** | Bacteria | Proteobacteria | Gammaproteobacteria | Xanthomonadales | Sinobacteraceae | Not assigned |
| **New.0.CleanUp.ReferenceOTU3078** | Bacteria | Proteobacteria | Gammaproteobacteria | Xanthomonadales | Sinobacteraceae | Not assigned |
| **New.0.CleanUp.ReferenceOTU8268** | Bacteria | Proteobacteria | Alphaproteobacteria | Sphingomonadales | Sphingomonadaceae | *Kaistobacter* |

Table S6

| **OTU ID** | **Kingdom** | **Phylum** | **Class** | **Order** | **Family** | **Genus** |
| --- | --- | --- | --- | --- | --- | --- |
| **811449** | Bacteria | Proteobacteria | Alphaproteobacteria | Caulobacterales | Caulobacteraceae | Not assigned |
| **689696** | Bacteria | Proteobacteria | Betaproteobacteria | Burkholderiales | Comamonadaceae | *Methylibium* |
| **211171** | Bacteria | Proteobacteria | Betaproteobacteria | Burkholderiales | Comamonadaceae | Not assigned |
| **849118** | Bacteria | Proteobacteria | Alphaproteobacteria | Rhizobiales | Hyphomicrobiaceae | *Devosia* |
| **850572** | Bacteria | Proteobacteria | Alphaproteobacteria | Rhizobiales | Rhizobiaceae | *Agrobacterium* |

Table S7A

| **Kingdom** | **Phylum** | **Class** | **Order** | **Family** | **p-value** | **RA high LUI (%)** | **RA low LUI (%)** |
| --- | --- | --- | --- | --- | --- | --- | --- |
| **Bacteria** | **Firmicutes** | **Bacilli** | **Turicibacterales** | **Turicibacteraceae** | **0.022** | **0.018** | **0.002** |
| **Bacteria** | **Proteobacteria** | **Alphaproteobacteria** | **Rhizobiales** | **Other** | **0.030** | **0.049** | **0.182** |
| **Bacteria** | **Acidobacteria** | **Solibacteres** | **Solibacterales** | **[Bryobacteraceae]** | **0.033** | **0.016** | **0.093** |
| **Bacteria** | **Bacteroidetes** | **Cytophagia** | **Cytophagales** | **Cytophagaceae** | **0.035** | **0.194** | **0.395** |
| Bacteria | Proteobacteria | Deltaproteobacteria | [Entotheonellales] | [Entotheonellaceae] | *0.054* | 0.003 | 0.035 |
| Bacteria | Proteobacteria | Alphaproteobacteria | Rhizobiales | Not assigned | *0.060* | 0.098 | 0.367 |
| Bacteria | Firmicutes | Bacilli | Bacillales | Not assigned | *0.064* | 0.019 | 0.052 |
| Bacteria | Proteobacteria | Gammaproteobacteria | Xanthomonadales | Sinobacteraceae | *0.065* | 1.978 | 7.375 |
| Bacteria | Actinobacteria | Actinobacteria | Actinomycetales | Actinosynnemataceae | *0.070* | 0.030 | 0.001 |
| Bacteria | Proteobacteria | Gammaproteobacteria | HTCC2188 | HTCC2089 | *0.076* | 0.002 | 0.012 |
| Bacteria | Actinobacteria | Thermoleophilia | Solirubrobacterales | Conexibacteraceae | *0.077* | 0.002 | 0.015 |
| Bacteria | Acidobacteria | Solibacteres | Solibacterales | Solibacteraceae | *0.080* | 0.149 | 0.427 |
| Bacteria | Bacteroidetes | VC2_1_Bac22 | Not assigned | Not assigned | *0.083* | 0.006 | 0.038 |
| Bacteria | Proteobacteria | Gammaproteobacteria | Legionellales | Legionellaceae | *0.088* | 0.034 | 0.000 |
| Bacteria | Proteobacteria | Betaproteobacteria | Methylophilales | Methylophilaceae | *0.092* | 0.185 | 0.028 |
| Bacteria | Proteobacteria | Deltaproteobacteria | Bdellovibrionales | Bacteriovoracaceae | *0.098* | 0.002 | 0.016 |
| Bacteria | Proteobacteria | Deltaproteobacteria | Myxococcales | Not assigned | *0.099* | 0.337 | 0.808 |
| Bacteria | Proteobacteria | Betaproteobacteria | SC-I-84 | Not assigned | *0.099* | 0.134 | 0.390 |

Table S7B

| **Kingdom** | **Phylum** | **Class** | **Order** | **Family** | **p-value** | **RA high LUI (%)** | **RA low LUI (%)** |
| --- | --- | --- | --- | --- | --- | --- | --- |
| **Bacteria** | **Proteobacteria** | **Deltaproteobacteria** | **Myxococcales** | **Cystobacteraceae** | **0.0003** | **0.095** | **0.205** |
| **Bacteria** | **Nitrospirae** | **Nitrospira** | **Nitrospirales** | **Nitrospiraceae** | **0.0003** | **1.356** | **0.200** |
| **Bacteria** | **Proteobacteria** | **Alphaproteobacteria** | **Not assigned** | **Not assigned** | **0.0010** | **0.082** | **0.257** |
| **Bacteria** | **Proteobacteria** | **Alphaproteobacteria** | **Rhizobiales** | **Not assigned** | **0.0022** | **0.648** | **0.953** |
| **Bacteria** | **Proteobacteria** | **Alphaproteobacteria** | **Rhodospirillales** | **Rhodospirillaceae** | **0.0022** | **1.712** | **2.384** |
| **Bacteria** | **Proteobacteria** | **Betaproteobacteria** | **Ellin6067** | **Not assigned** | **0.0028** | **2.966** | **4.321** |
| **Bacteria** | **Proteobacteria** | **Betaproteobacteria** | **Nitrosomonadales** | **Nitrosomonadaceae** | **0.0042** | **0.082** | **0.007** |
| **Bacteria** | **Proteobacteria** | **TA18** | **PHOS-HD29** | **Not assigned** | **0.0042** | **0.012** | **0.003** |
| **Bacteria** | **Proteobacteria** | **Alphaproteobacteria** | **Ellin329** | **Not assigned** | **0.0050** | **0.307** | **0.617** |
| **Bacteria** | **Proteobacteria** | **Alphaproteobacteria** | **Rhodobacterales** | **Hyphomonadaceae** | **0.0062** | **0.237** | **0.417** |
| **Bacteria** | **Proteobacteria** | **Alphaproteobacteria** | **Rhodobacterales** | **Rhodobacteraceae** | **0.0066** | **0.351** | **0.159** |
| **Bacteria** | **Proteobacteria** | **Betaproteobacteria** | **IS-44** | **Not assigned** | **0.0227** | **0.074** | **0.246** |
| **Bacteria** | **Actinobacteria** | **Actinobacteria** | **Actinomycetales** | **Propionibacteriaceae** | **0.0230** | **1.206** | **0.470** |
| **Bacteria** | **Proteobacteria** | **Betaproteobacteria** | **Rhodocyclales** | **Rhodocyclaceae** | **0.0244** | **0.314** | **0.133** |
| **Bacteria** | **Tenericutes** | **Mollicutes** | **Anaeroplasmatales** | **Anaeroplasmataceae** | **0.0334** | **0.001** | **0.010** |
| **Bacteria** | **Proteobacteria** | **Alphaproteobacteria** | **Rhizobiales** | **Beijerinckiaceae** | **0.0356** | **0.038** | **0.022** |
| **Bacteria** | **Acidobacteria** | **Acidobacteriia** | **Acidobacteriales** | **Acidobacteriaceae** | **0.0358** | **0.002** | **0.012** |
| **Bacteria** | **Actinobacteria** | **Actinobacteria** | **Actinomycetales** | **Intrasporangiaceae** | **0.0440** | **0.047** | **0.020** |
| **Bacteria** | **Proteobacteria** | **Alphaproteobacteria** | **Rickettsiales** | **Not assigned** | **0.0461** | **0.015** | **0.026** |
| Bacteria | Firmicutes | Clostridia | Clostridiales | Clostridiaceae | *0.0532* | 0.124 | 0.049 |
| Bacteria | Proteobacteria | Gammaproteobacteria | Chromatiales | Not assigned | *0.0550* | 0.002 | 0.010 |
| Bacteria | Actinobacteria | Actinobacteria | Actinomycetales | Pseudonocardiaceae | *0.0567* | 0.434 | 0.200 |
| Bacteria | Proteobacteria | Alphaproteobacteria | Rhodospirillales | Not assigned | *0.0575* | 0.182 | 0.083 |
| Bacteria | Proteobacteria | Gammaproteobacteria | Other | Other | *0.0580* | 0.119 | 0.231 |
| Bacteria | Acidobacteria | Solibacteres | Solibacterales | Not assigned | *0.0630* | 1.054 | 1.427 |
| Bacteria | Actinobacteria | Actinobacteria | Actinomycetales | Streptosporangiaceae | *0.0747* | 0.012 | 0.043 |
| Bacteria | Proteobacteria | Gammaproteobacteria | Thiotrichales | Piscirickettsiaceae | *0.0753* | 0.251 | 0.528 |
| Bacteria | Bacteroidetes | Sphingobacteriia | Sphingobacteriales | Sphingobacteriaceae | *0.0801* | 1.223 | 0.509 |
| Bacteria | Proteobacteria | Betaproteobacteria | Burkholderiales | Alcaligenaceae | *0.0812* | 0.554 | 1.363 |
| Bacteria | Actinobacteria | Actinobacteria | Actinomycetales | Frankiaceae | *0.0815* | 0.184 | 0.054 |
| Bacteria | Bacteroidetes | Flavobacteriia | Flavobacteriales | Cryomorphaceae | *0.0833* | 0.117 | 0.049 |
| Bacteria | Acidobacteria | DA052 | Ellin6513 | Not assigned | *0.0873* | 0.000 | 0.020 |
| Bacteria | Chloroflexi | Anaerolineae | SHA-20 | Not assigned | *0.0875* | 0.002 | 0.012 |
| Bacteria | Proteobacteria | Betaproteobacteria | Not assigned | Not assigned | *0.0918* | 1.575 | 2.388 |
| Bacteria | Proteobacteria | Deltaproteobacteria | Not assigned | Not assigned | *0.0941* | 0.019 | 0.049 |
| Bacteria | Proteobacteria | Gammaproteobacteria | [Marinicellales] | [Marinicellaceae] | *0.0960* | 0.073 | 0.301 |
| Bacteria | TM6 | SJA-4 | Not assigned | Not assigned | *0.0985* | 0.025 | 0.107 |
| Bacteria | Proteobacteria | Deltaproteobacteria | [Entotheonellales] | [Entotheonellaceae] | *0.0992* | 0.175 | 0.421 |

Table S7C

| **Kingdom** | **Phylum** | **Class** | **Order** | **Family** | **p-value** | **RA high LUI (%)** | **RA low LUI(%)** |
| --- | --- | --- | --- | --- | --- | --- | --- |
| **Bacteria** | **Proteobacteria** | **Betaproteobacteria** | **A21b** | **UD5** | **0.0002** | **0.422** | **0.167** |
| **Bacteria** | **Proteobacteria** | **Deltaproteobacteria** | **Desulfuromonadales** | **Geobacteraceae** | **0.0002** | **0.366** | **0.015** |
| **Bacteria** | **Proteobacteria** | **Alphaproteobacteria** | **Ellin329** | **Not assigned** | **0.0002** | **0.200** | **0.401** |
| **Bacteria** | **Elusimicrobia** | **Elusimicrobia** | **FAC88** | **Not assigned** | **0.0003** | **0.024** | **0.004** |
| **Bacteria** | **Proteobacteria** | **Alphaproteobacteria** | **Rhodobacterales** | **Rhodobacteraceae** | **0.0003** | **0.141** | **0.034** |
| **Bacteria** | **Actinobacteria** | **Actinobacteria** | **Actinomycetales** | **Intrasporangiaceae** | **0.0013** | **0.031** | **0.003** |
| **Bacteria** | **Proteobacteria** | **Gammaproteobacteria** | **Alteromonadales** | **125ds10** | **0.0015** | **0.027** | **0.077** |
| **Bacteria** | **Nitrospirae** | **Nitrospira** | **Nitrospirales** | **Nitrospiraceae** | **0.0016** | **1.395** | **0.212** |
| **Bacteria** | **OD1** | **ABY1** | **Not assigned** | **Not assigned** | **0.0016** | **0.011** | **0.000** |
| **Bacteria** | **Proteobacteria** | **Alphaproteobacteria** | **Rhodospirillales** | **Rhodospirillaceae** | **0.0020** | **1.528** | **2.585** |
| **Bacteria** | **Proteobacteria** | **Betaproteobacteria** | **Nitrosomonadales** | **Nitrosomonadaceae** | **0.0022** | **0.121** | **0.005** |
| **Bacteria** | **Proteobacteria** | **Alphaproteobacteria** | **Rhizobiales** | **Not assigned** | **0.0025** | **0.710** | **0.975** |
| **Bacteria** | **Proteobacteria** | **Gammaproteobacteria** | **Alteromonadales** | **211ds20** | **0.0033** | **0.032** | **0.005** |
| **Bacteria** | **Firmicutes** | **Clostridia** | **Clostridiales** | **Peptostreptococcaceae** | **0.0050** | **0.034** | **0.006** |
| **Bacteria** | **Firmicutes** | **Clostridia** | **Clostridiales** | **Clostridiaceae** | **0.0053** | **0.285** | **0.082** |
| **Bacteria** | **Chloroflexi** | **Ellin6529** | **Not assigned** | **Not assigned** | **0.0056** | **0.046** | **0.019** |
| **Bacteria** | **Actinobacteria** | **MB-A2-108** | **0319-7L14** | **Not assigned** | **0.0063** | **0.065** | **0.018** |
| **Bacteria** | **Actinobacteria** | **Actinobacteria** | **Actinomycetales** | **Micromonosporaceae** | **0.0072** | **0.228** | **0.507** |
| **Bacteria** | **Proteobacteria** | **Deltaproteobacteria** | **NB1-j** | **NB1-i** | **0.0073** | **0.168** | **0.417** |
| **Bacteria** | **Planctomycetes** | **C6** | **d113** | **Not assigned** | **0.0074** | **0.011** | **0.000** |
| **Bacteria** | **Proteobacteria** | **Deltaproteobacteria** | **Myxococcales** | **Not assigned** | **0.0106** | **2.976** | **4.215** |
| **Bacteria** | **Bacteroidetes** | **Bacteroidia** | **Bacteroidales** | **Not assigned** | **0.0109** | **0.009** | **0.000** |
| **Bacteria** | **Actinobacteria** | **Actinobacteria** | **Actinomycetales** | **Streptosporangiaceae** | **0.0123** | **0.011** | **0.111** |
| **Bacteria** | **Actinobacteria** | **Actinobacteria** | **Actinomycetales** | **Propionibacteriaceae** | **0.0160** | **0.832** | **0.417** |
| **Bacteria** | **Proteobacteria** | **Deltaproteobacteria** | **Myxococcales** | **Cystobacteraceae** | **0.0175** | **0.100** | **0.280** |
| **Bacteria** | **Nitrospirae** | **Nitrospira** | **Nitrospirales** | **FW** | **0.0187** | **0.044** | **0.000** |
| **Bacteria** | **Proteobacteria** | **Deltaproteobacteria** | **Spirobacillales** | **Not assigned** | **0.0210** | **0.008** | **0.001** |
| **Bacteria** | **Proteobacteria** | **Alphaproteobacteria** | **Rhizobiales** | **Xanthobacteraceae** | **0.0215** | **0.064** | **0.103** |
| **Bacteria** | **TM6** | **SJA-4** | **Not assigned** | **Not assigned** | **0.0225** | **0.090** | **0.308** |
| **Bacteria** | **Planctomycetes** | **Other** | **Other** | **Other** | **0.0229** | **0.014** | **0.047** |
| **Bacteria** | **Actinobacteria** | **Thermoleophilia** | **Gaiellales** | **Gaiellaceae** | **0.0243** | **1.687** | **1.000** |
| **Bacteria** | **Acidobacteria** | **Solibacteres** | **Solibacterales** | **AKIW659** | **0.0263** | **0.048** | **0.012** |
| **Bacteria** | **Gemmatimonadetes** | **Gemmatimonadetes** | **Ellin5290** | **Not assigned** | **0.0279** | **0.041** | **0.195** |
| **Bacteria** | **Proteobacteria** | **Deltaproteobacteria** | **Myxococcales** | **Polyangiaceae** | **0.0291** | **0.252** | **0.353** |
| **Bacteria** | **Spirochaetes** | **[Leptospirae]** | **[Leptospirales]** | **Leptospiraceae** | **0.0295** | **0.056** | **0.030** |
| **Bacteria** | **Bacteroidetes** | **Sphingobacteriia** | **Sphingobacteriales** | **Sphingobacteriaceae** | **0.0301** | **0.204** | **0.064** |
| **Bacteria** | **Actinobacteria** | **Actinobacteria** | **Actinomycetales** | **Pseudonocardiaceae** | **0.0324** | **0.281** | **2.107** |
| **Bacteria** | **Chloroflexi** | **TK17** | **mle1-48** | **Not assigned** | **0.0354** | **0.019** | **0.006** |
| **Bacteria** | **Acidobacteria** | **EC1113** | **Not assigned** | **Not assigned** | **0.0357** | **0.014** | **0.005** |
| **Bacteria** | **Gemmatimonadetes** | **Gemmatimonadetes** | **Gemmatimonadales** | **Ellin5301** | **0.0378** | **0.006** | **0.098** |
| **Bacteria** | **Proteobacteria** | **Alphaproteobacteria** | **Rhizobiales** | **Beijerinckiaceae** | **0.0389** | **0.051** | **0.032** |
| **Bacteria** | **Proteobacteria** | **Betaproteobacteria** | **Rhodocyclales** | **Rhodocyclaceae** | **0.0403** | **0.336** | **0.092** |
| **Bacteria** | **WS3** | **PRR-12** | **Sediment-1** | **Not assigned** | **0.0408** | **0.036** | **0.010** |
| **Bacteria** | **Actinobacteria** | **Acidimicrobiia** | **Acidimicrobiales** | **AKIW874** | **0.0429** | **0.366** | **0.774** |
| **Bacteria** | **Actinobacteria** | **Actinobacteria** | **Actinomycetales** | **Other** | **0.0432** | **0.133** | **0.227** |
| **Bacteria** | **Firmicutes** | **Bacilli** | **Turicibacterales** | **Turicibacteraceae** | **0.0448** | **0.021** | **0.005** |
| **Bacteria** | **Proteobacteria** | **Betaproteobacteria** | **Burkholderiales** | **Oxalobacteraceae** | **0.0455** | **0.648** | **0.267** |
| **Bacteria** | **Proteobacteria** | **Betaproteobacteria** | **Methylophilales** | **Methylophilaceae** | **0.0487** | **0.167** | **0.036** |
| Bacteria | Chlorobi | OPB56 | Not assigned | Not assigned | *0.0502* | 0.024 | 0.009 |
| Bacteria | Planctomycetes | Not assigned | Not assigned | Not assigned | *0.0503* | 0.032 | 0.064 |
| Bacteria | Spirochaetes | Spirochaetes | Spirochaetales | Spirochaetaceae | *0.0526* | 0.008 | 0.021 |
| Bacteria | Bacteroidetes | Sphingobacteriia | Sphingobacteriales | Not assigned | *0.0536* | 2.927 | 1.546 |
| Bacteria | Acidobacteria | Solibacteres | Solibacterales | Not assigned | *0.0580* | 1.353 | 2.139 |
| Bacteria | Chloroflexi | Anaerolineae | SBR1031 | A4b | *0.0611* | 0.034 | 0.082 |
| Bacteria | Chlamydiae | Chlamydiia | Chlamydiales | Simkaniaceae | *0.0645* | 0.006 | 0.031 |
| Bacteria | Proteobacteria | Deltaproteobacteria | Bdellovibrionales | Bdellovibrionaceae | *0.0680* | 0.027 | 0.015 |
| Bacteria | Acidobacteria | DA052 | Ellin6513 | Not assigned | *0.0693* | 0.002 | 0.023 |
| Bacteria | WS3 | PRR-12 | LD1-PA13 | Not assigned | *0.0696* | 0.046 | 0.143 |
| Bacteria | Proteobacteria | Alphaproteobacteria | Rhizobiales | Rhizobiaceae | *0.0785* | 0.194 | 0.104 |
| Bacteria | WS3 | PRR-12 | Sediment-1 | PRR-10 | *0.0799* | 0.003 | 0.011 |
| Bacteria | Proteobacteria | Betaproteobacteria | SC-I-84 | Not assigned | *0.0818* | 2.586 | 3.524 |
| Bacteria | Proteobacteria | Deltaproteobacteria | Myxococcales | Myxococcaceae | *0.0831* | 0.020 | 0.000 |
| Bacteria | Acidobacteria | Solibacteres | Solibacterales | Solibacteraceae | *0.0844* | 1.598 | 2.384 |
| Bacteria | Proteobacteria | Deltaproteobacteria | Myxococcales | Nannocystaceae | *0.0851* | 0.077 | 0.036 |
| Bacteria | GN02 | 3BR-5F | Not assigned | Not assigned | *0.0857* | 0.009 | 0.000 |
| Bacteria | Proteobacteria | Deltaproteobacteria | [Entotheonellales] | [Entotheonellaceae] | *0.0878* | 0.366 | 0.597 |
| Bacteria | Actinobacteria | Actinobacteria | Actinomycetales | Nakamurellaceae | *0.0896* | 0.070 | 0.038 |
| Bacteria | Gemmatimonadetes | Gemm-5 | Not assigned | Not assigned | *0.0916* | 0.008 | 0.031 |
| Bacteria | Chlorobi | SJA-28 | Not assigned | Not assigned | *0.0932* | 0.871 | 0.284 |
| Bacteria | Proteobacteria | Betaproteobacteria | Other | Other | *0.0998* | 0.046 | 0.024 |
